# Clinical Characteristics and Outcomes of ICU Patients During the First Post-COVID-19 2023–2024 Influenza Season in The Netherlands

**DOI:** 10.3390/v17111467

**Published:** 2025-11-01

**Authors:** Sjoerd van der Bie, Johannes P. C. van den Akker, Ramon C. Fluit, Steven F. L. van Lelyveld, Maarten E. Nuver, Suzanne Stads, Peter Spronk, Carina Bethlehem, Romy Takken, Corstiaan A. den Uil, Jantine Van Holten, Rutger Van Raalte, Jurre Kuipers, Marc Schluep, Matty Koopmans, Louise Urlings-Strop, Esther K. Haspels-Hogervorst, Nina E. Disseldorp, Jan Elderman, Roy Sneijder, Jasper de Roos, Merijn Kant, Robbert G. Bentvelsen, Tobias Neijzen, Dorien Kiers, Klaas de Groot, Ashley de Bie, Peter de Jager, Michiel Blans, Myrthe de Haas, Mariska Lont, Stephanie Koster, Angelique C. M. Jansen, Petronella E. Deetman, Fieke Mus, Ralph Nowitzky, Lucas Brands, Hazra Moeniralam, Erik Schaftenaar, Martijn van Tellingen, Jasper Haringman, Emily Thieme Groen, Lenneke E. M. Haas, Wouter de Ruijter, Rob Wilting, Hetty Kranen, Charlotte H. S. B. van den Berg, Diederik Gommers, Evert-jan Wils, Henrik Endeman, Marco Goeijenbier

**Affiliations:** 1 Department of Intensive Care Medicine, Spaarne Gasthuis, 2035 RC Haarlem Hoofddorp, The Netherlands; svanderbie@spaarnegasthuis.nl (S.v.d.B.); rfluit@spaarnegasthuis.nl (R.C.F.); 2 Department of Intensive Care Medicine, Erasmus MC, 3015 GD Rotterdam, The Netherlands; j.vandenakker@erasmusmc.nl (J.P.C.v.d.A.);; 3 Department of Internal Medicine, Spaarne Gasthuis, 2035 RC Haarlem, The Netherlands; s.van.lelyveld@spaarnegasthuis.nl; 4 Department of Intensive Care, Meander Medisch Centrum, 3813 TZ Amersfoort, The Netherlands; me.nuver@meandermc.nl; 5 Department of Intensive Care, Ikazia, 3083 AN Rotterdam, The Netherlands; 6 Department of Intensive Care, Gelre Hospital, 7334 DZ Apeldoorn, The Netherlands; 7 Department of Intensive Care, Medisch Centrum Leeuwarden, 8934 AD Leeuwarden, The Netherlands; 8 Department of Intensive Care, Maasstad Ziekenhuis, 3079 DZ Rotterdam, The Netherlandsuilc@maasstadziekenhuis.nl (C.A.d.U.);; 9 Department of Intensive Care, Tergooi Medisch Centrum, 1212 VG Hilversum, The Netherlands; 10 Department of Intensive Care, Elisabeth-TweeSteden Ziekenhuis, 5042 AD Tilburg, The Netherlandsr.wilting@etz.nl (R.W.); 11 Department of Intensive Care, Bravis Ziekenhuis, 4624 VT Bergen op Zoom, The Netherlands; 12 Department of Intensive Care, Onze Lieve Vrouwe Gasthuis, 1091 AC Amsterdam, The Netherlands; 13 Department of Intensive Care, Reinier de Graaf Groep, 2625 AD Delft, The Netherlands; l.urlings@rdgg.nl; 14 Intensive Care Department, Martini Hospital, 9728 NT Groningen, The Netherlands; 15 Intensive Care Department, IJsselland Ziekenhuis, 2906 ZC Capelle aan den IJssel, The Netherlands; 16 Intensive Care Department, Admiraal de Ruyter Ziekenhuis, 4462 RA Goes, The Netherlands; 17 Department of Intensive Care, Amphia Hospital, 4818 CK Breda, The Netherlands; 18 Microvida Laboratory for Microbiology, Amphia Hospital, 4818 CK Breda, The Netherlands; 19 Medische Faculteit, Amsterdam UMC, Locatie Vrije Universiteit Medisch Centrum, 1081 BT Amsterdam, The Netherlands; 20 Department of Intensive Care, Franciscus Gasthuis & Vlietland, 3045 PM Rotterdam, The Netherlandse.wils@franciscus.nl (E.-j.W.); 21 Department of Intensive Care, Maxima Medisch Centrum, 5631 BM Eindhoven, The Netherlands; 22 Department of Intensive Care, Catharina Ziekenhuis, 5623 EJ Eindhoven, The Netherlands; 23 Department of Intensive Care, Jeroen Bosch Ziekenhuis, 5223 GZ ‘s-Hertogenbosch, The Netherlands; 24 Department of Intensive Care, Rijnstate, 6815 AD Arnhem, The Netherlands; mblans@rijnstate.nl (M.B.);; 25 Department of Intensive Care, Dijklander Ziekenhuis, 1624 NP Hoorn, The Netherlands; 26 Department of Intensive Care, Zaans Medisch Centrum, 1502 DV Zaandam, The Netherlands; 27 Department of Intensive Care, Alrijne Ziekenhuis, 2334 CK Leiden, The Netherlands; 28 Department of Intensive Care, Albert Schweitzer Ziekenhuis, 3318 AT Dordrecht, The Netherlands; p.e.deetman@asz.nl; 29 Department of Intensive Care, HagaZiekenhuis, 2545 AA Den Haag, The Netherlands; 30 Department of Intensive Care, Groene Hart Ziekenhuis, 2803 HH Gouda, The Netherlands; 31 Department of Intensive Care, St Antonius Ziekenhuis, 3435 CM Nieuwegein, The Netherlandse.schaftenaar@antoniusziekenhuis.nl (E.S.); 32 Department of Intensive Care, Frisius MC, 8934 AD Leeuwarden, The Netherlands; 33 Department of Intensive Care, Isala, 8025 AB Zwolle, The Netherlands; j.haringman@isala.nl; 34 Department of Intensive Care, Flevoziekenhuis, 1315 RA Almere, The Netherlands; 35 Department of Intensive Care, Diakonessenhuis, 3582 KE Utrecht, The Netherlands; lvlelyveld@diakhuis.nl; 36 Department of Intensive Care, Noordwest Ziekenhuisgroep, 1815 JD Alkmaar, The Netherlands; 37 Department of Intensive Care, Universitair Medisch Centrum Groningen, 9713 GZ Groningen, The Netherlands

**Keywords:** influenza, ARDS, respiratory failure, viral pneumonia

## Abstract

Background: Influenza can cause severe complications, especially in patients with specific risk factors or comorbidities associated with poor outcomes. Some patients are at increased risk of a complicated disease course, including secondary infections, ICU admission, and the need for mechanical ventilation. The first post–COVID-19 seasonal influenza season placed a substantial burden on Dutch ICUs. This study investigates the disease course and outcomes of ICU patients with influenza. Methods: A retrospective influenza registry study was conducted across 34 Dutch ICUs, including patients aged 18 and older admitted to the ICU with a positive influenza RT-PCR test, between 1 November 2023 and 17 March 2024. Data on demographic information, medical history, clinical symptoms, laboratory and imaging results, parameters of mechanical ventilation, additional treatments, length of hospital stay, and mortality was retrieved from the electronic patient record. Results: A total of 498 patients were included in the study. The median age was 64 (IQR: 55–72) years and 58.8% of the patients were men. The most common comorbidities were cardiovascular disease (34.1%), chronic obstructive pulmonary disease (COPD) (31.5%), and diabetes (22.3%). Bacterial co-infections were present in 37.6% of the patients. Invasive mechanical ventilation (IMV) was necessary in 46.0% of patients, 38.0% of those requiring IMV were treated in prone position. A substantial mortality rate was observed, with an ICU mortality rate of 21.9% and an additional hospital mortality rate of 5.2%. Conclusion: This study described the characteristics and course of disease of all patients with laboratory-confirmed influenza infection admitted to one of the 34 participating Dutch ICUs between November 2023 and March 2024. The major findings of this study are the substantial mortality rate, a high proportion of patients with bacterial co-infections, and a significant percentage of patients requiring IMV and prone position ventilation. Finally, patients without comorbidities that were admitted to the ICU with an influenza virus infection showed severe disease parameters but had a lower mortality than patients with comorbidities.

## 1. Introduction

Influenza can cause illness ranging from mild to severe, with the majority of patients experiencing only mild respiratory symptoms. However, others, particularly the elderly and those with underlying chronic health conditions, are at risk of severe illness such as pneumonia, sepsis, or respiratory failure, often requiring ICU admission and mechanical ventilation [1,2,3,4,5]. The incidence of influenza exhibits seasonal variation, typically peaking in the Netherlands between December and March [6]. However, this pattern was disrupted during the SARS-CoV-2 (COVID-19) pandemic, where for at least two years hardly any influenza infections were noted [7]. After the pandemic, seasonality of respiratory pathogens reoccurred with a change in secondary infections like group A streptococci, atypical infections such as *Mycoplasma pneumoniae* and possible rebound infections with influenza A after the cessation of the preventive non-pharmaceutical interventions (NPIs) [8,9]. The influenza A[H1N1]pdm09 and SARS-CoV-2 pandemics have highlighted the potentially disastrous course of community acquired viral pneumonia. Additionally, due to the high incidence of 20% among patients with acute respiratory infections and a high mortality of 7%, influenza remains to impose a significant annual burden on both patients and healthcare systems, despite annual vaccination campaigns [1,3].

To manage respiratory distress and prevent further complications, ICU admission is required to provide advanced medical supportive care, such as high flow oxygen therapy, mechanical ventilation or extracorporeal membrane oxygenation (ECMO) [10,11,12]. In addition to respiratory support, some patients receive antibiotics and/or antiviral medications, such as oseltamivir [13]. Nevertheless, despite these escalating treatments in the ICU, the mortality rate for influenza patients admitted to the ICU is approximately 38% [11]. Alongside an increase in comorbidities that make patients more vulnerable to severe infections, this mortality rate rise may be partly attributed to influenza-associated pulmonary co-infections or secondary infections or viral reactivations. Previous studies have demonstrated that influenza patients with co-infections are at a heightened risk of invasive mechanical ventilation (IMV), as well as an increased mortality risk [14,15]. The most common bacterial co-infections in patients with influenza are *Staphylococcus aureus*, *Streptococcus pneumoniae* and *Klebsiella pneumoniae*. Furthermore, infections with fungal pathogens as *Aspergillus fumigatus* are frequently reported as well [11,16]. Therefore, guidelines suggests routinely testing for concomitant *Aspergillus* infections in complicated influenza cases, and even start pre-emptive antifungal therapy before test results are known based on the POSA-FLU study [17]. These infections may complicate the course of treatment and lead to the higher mortality and burden of disease found in patients admitted to the ICU.

Since the COVID-19 pandemic, clinical research on severe acute respiratory infections (SARIs) has largely focused on SARS-CoV-2. Nevertheless, influenza continues to be a leading cause of ICU admissions during seasonal peaks. To date, influenza has not been extensively investigated across intensive care units nationwide in the Netherlands. Although the 2023–2024 influenza season in the Netherlands was relatively short, clinicians reported a considerable burden on ICUs [18]. This study aims to conduct an in-depth case analysis to describe the characteristics and course of disease as well as the outcomes of the first influenza season in multiple nationwide ICUs since the onset of the COVID-19 pandemic.

## 2. Materials and Methods

### 2.1. Study Design

A retrospective study was conducted including all patients admitted to an ICU with a positive real-time reverse transcriptase polymerase chain reaction (RT-PCR) for influenza in any respiratory sample. These data were collected between 1 November 2023 and 17 March 2024, in one of 34 participating ICUs in the Netherlands. The list of participating ICUs is presented in Appendix A. The total capacity of these ICUs was 545 beds, consisting of 55% of the total number of ICU beds in the Netherlands. This study was approved by the Institutional Review Board of the Spaarne Gasthuis, the Netherlands (2024.0024), as well as by the local hospital review committees of all participating hospitals.

### 2.2. Inclusion Criteria

All patients 18 years of age and older, admitted to the ICU with a positive influenza virus sample before or during admission to the ICU were included. A positive sample was defined as the detection of influenza A or B RNA through real-time RT-PCR in any respiratory sample, including nasopharyngeal/oropharyngeal (NP/OP) swab, sputum or bronchial alveolar lavage (BAL). In order to obtain a complete and total view of ICU resources used in this period of time, no exclusion criteria were used.

### 2.3. Data Collection

Before the start of data collection, patients were provided with an information letter allowing them to opt-out of the study if they did not consent to the use of their data. If patients did not opt-out within 2 weeks, their data was collected from the electronic patient files. The data collection involved retrieving clinical data from patient records including demographic information, medical history, clinical symptoms, laboratory and imaging results, parameters of mechanical ventilation, additional treatments, length of hospital stay, and mortality. Data were captured, managed and stored using ResearchManager (ResearchManager B.V., Deventer, The Netherlands; https://my-researchmanager.com, accessed on 01 October 2023).

### 2.4. Variables and Definitions

Cardiovascular comorbidities included all types of cardiovascular diseases such as hypertension, atrial fibrillation myocardial infarction and hearth failure. Galactomannan enzyme-linked immunosorbent assay (ELISA) tests results on BAL fluid or serum were considered positive in case of a value of 0.8 or higher. Mortality was categorized as ICU mortality and hospital mortality. The use of immunosuppressants included systemically administered corticosteroids, either orally or intravenously, and excluded inhaled corticosteroids. Co-infection was defined as the detection of an additional bacterial or fungal pathogen in any of the aforementioned respiratory samples, combined with the treating physician’s assessment, as noted in the patient record, that it constituted a co-infection and warranted treatment. P/F ratios in patients receiving non-invasive respiratory support (NIRS) were calculated using the FiO_2_ provided by the NIRS device, although this method has not been formally validated for this purpose.

### 2.5. Data Analysis

As this is a registry-based study, the primary outcome was to describe the demographic characteristics, clinical outcomes, treatment and disease course of the cohort. Secondary outcomes included the identification of risk factors associated with increased mortality, as well as comparisons of the clinical course between patients with and without comorbidities, and between those with and without bacterial co-infections. Demographic parameters, clinical outcomes, and additional test results are presented as categorical variables with percentages or continuous variables with mean (±SD) or median (interquartile range, IQR), depending on normality. Given the non-normal distribution of most variables, group differences were assessed using the Mann–Whitney U test for continuous variables and the Chi-square test for dichotomous variables. Viral co-detections, such as rhinovirus, were excluded due to the risk of bias.

Associations were examined using regression analyses: logistic regression for dichotomous outcomes and linear regression for continuous outcomes. Candidate predictors were selected based on univariate regression analyses with a *p*-value threshold of 0.3, followed by a backward selection strategy in multivariate models, excluding variables with the least significant *p*-value greater than 0.05, until only statistically significant predictors remained in the final model. To minimize the risk of overfitting and false-positive associations, the number of predictors included in the final model adhered to the commonly accepted rule of at least 10 events per variable for logistic regression. Predictors considered for inclusions encompassed of baseline characteristics, co-morbidities and laboratory results.

As this was a descriptive study, analyses were conducted using available data only. Participants with missing values on specific variables were excluded from the corresponding analyses. No formal assessment of the pattern or mechanism of missing data was performed. However, total missing data was considered low, except for vaccination status.

Statistical analyses were conducted using SPSS version 26.

## 3. Results

### 3.1. Baseline Characteristics and Co-Morbidities

A total of 498 patients with a positive influenza virus RT-PCR were included in this study. Most patients were admitted for respiratory failure caused by pneumonia (54.0%) or sepsis (9.2%).

The median age of patients at admission was 64 (IQR 55–72) years and 58.8% were men. For the majority of patients (94%), influenza vaccination status was unknown. Baseline characteristics are shown in Table 1. The most common comorbidities at admission were cardiovascular diseases (34.1%), chronic obstructive pulmonary disease (COPD) (31.5%), and diabetes (type 1 or 2) (22.3%). Immunosuppressant use was common (10.6%). 66.7% of the patients did not have treatment restrictions. A cardiopulmonary resuscitation (CPR) restriction was present in 31.3% of patients, and 18.5% either declined or were deemed unsuitable for IMV.

The median Sequential Organ Failure Assessment (SOFA) score at admission was 6 (IQR 3–6), while the median Simplified Acute Physiology II score (SAPS II) was 36 (IQR 27–48). Laboratory testing revealed that the median leukocyte count was 10.5 × 10^9^/L (IQR 6.2–14.8 × 10^9^/L), and the median CRP level was 137 mg/L (IQR 58–276 mg/L). The majority of patients had a P/F-ratio between 100 mmHg and 200 mmHg at ICU admission. The data on the disease severity scores and laboratory testing are shown in Table 1.

A substantial proportion of patients (26.1%) had no known comorbidities prior to ICU admission. A comparison was conducted to investigate differences between these groups that might contribute to course of disease. Patients without comorbidities were younger than those with comorbidities (57 years (IQR 24–64) vs. 67 years (IQR 59–74); *p* < 0.0001). Additionally, patients without comorbidities were more likely to have bacterial co-infections (60 (46.2%) vs. 119 (34.4%); *p* = 0.018) and more frequently exhibited consolidations on chest X-rays (99 (76.2%) vs. 198 (57.2%); *p* < 0.0001). Overall, patients without comorbidities were more frequently mechanically ventilated (76 (56.2%) vs. 149 (43.1%); *p* = 0.005), had a lower P/F-ratio (107 mmHg (IQR 71–150) vs. 122 mmHg (IQR 86–184); *p* = 0.009), were ventilated in prone position more frequently (40 (30.8%) vs. 44 (127%); *p* < 0.0001) and had a longer duration of ICU admission (5 days (IQR 2–13) vs. 3 days (IQR 1–7); *p* = 0.009). However, ICU and hospital mortality were lower in patients without comorbidities (ICU mortality: 18 (13.8%) vs. 88 (25.4%); *p* = 0.008; additional hospital mortality: 1 (0.8%) vs. 24 (6.9%), *p* = 0.008).

### 3.2. Co-Infections

During ICU admission, 37.6% of patients had a bacterial co-infection. The most common pathogens were Streptococcus pneumoniae (31.1%), Group A Streptococcus (20.3%), and Staphylococcus aureus (8.5%). Aspergillus species were cultured in respiratory samples 9.8% of patients.

Among mechanically ventilated patients, galactomannan was detected in BAL fluid (13.1%) and blood serum (3.9%). In 61% of the mechanically ventilated patients Aspergillus Galactomannan test was not performed. Data on co-infections are summarized in Table 2.

Patients with bacterial co-infections were younger (63 (IQR 55–69) vs. 65 (IQR 56–73) years, *p* = 0.015) and had fewer cardiovascular comorbidities (52 (27.8%) vs. 118 (37.9%), *p* = 0.021) when compared to the group without bacterial co-infections. They had higher SOFA scores (7 (IQR 4–10) vs. 5 (IQR 3–8), *p* = 0.002) and higher CRP levels (215 (IQR 99–333) vs. 102 (IQR 50–192) mg/L, *p* < 0.0001). Imaging showed more consolidations (142 (75.9%) vs. 162 (52.1%), *p* < 0.0001) and pleural effusions (37 (19.8%) vs. 37 (11.9%), *p* = 0.017). These patients had worse oxygenation on admission (P/F ratio: 101 (IQR 75–155) vs. 139 (IQR 94–192) mmHg, *p* < 0.001), were more likely to require IMV (74 (39.6%) vs. 89 (28.6%) *p* = 0.01) and underwent proning more frequently (51 (27.3%) vs. 36 (11.6%), *p* = 0.001). They more frequently received steroids (127 (67.9%) vs. 174 (55.9%), *p* = 0.008). Length of stay in the ICU was longer (4 (IQR 2–12) vs. 3 (IQR 1–8) days, *p* = 0.001), but total length of stay in the hospital stay as well as mortality rates did not differ between these two groups.

### 3.3. Diagnostic Imaging

Diagnostic imaging, including chest X-rays and/or computed tomography (CT) scans, revealed consolidative abnormalities in 61.0% of the X-rays and 83.0% of the CT scans, with the majority being bilateral (57%). Pleural effusions on chest X-ray was seen in 14.9%, and 24.8% on CT scan. The most common findings on CT scan were consolidations (83.0%), ground-glass opacity (34.7%), and pleural effusion (24.8%). All diagnostic imaging results are shown in Table 3.

### 3.4. Respiratory Support

More than half of the patients (59.2%) underwent NIRS at admission, including high-flow nasal oxygen therapy (HFOT) (58.6%), continuous positive airway pressure (CPAP) (12.3%), and bilevel positive airway pressure (BiPAP) (27.1%). The median duration of NIRS in the ICU was 1 day (IQR 1–2). This was partly explained by a substantial proportion of these patients (36.7%) experiencing progression of respiratory failure, necessitating escalation to IMV. At the time of escalation to IMV, 38.7% of patients had a P/F ratio below 100 mmHg, and another 38.7% had a P/F ratio between 100 mmHg and 200 mmHg. Most patients progressed from NIRS to IMV within one day of starting NIRS. The data on NIRS are displayed in Table 4.

Almost a quarter of the patients (24.3%) was directly intubated and mechanically ventilated upon admission to the ICU. Including those with NIRS failure who required escalation from NIRS to IMV, a total of 229 patients (46.0%) were mechanically ventilated. The median duration of mechanical ventilation was 6 days (IQR 3–17). Eighty percent of patients had a P/F ratio below 200 mmHg at the initiation of IMV, with the majority between 100 mmHg and 200 mmHg. The median lowest P/F ratio observed during admission was 119 mmHg (IQR 83–179). The median highest measured PaCO2 during the full course of mechanical ventilation was 55.1 (IQR 44.1–70.0) mmHg. Thirty eight percent of the patients underwent proning due to hypoxemic respiratory failure. Proning was predominantly initiated immediately after intubation, with a median duration of 2 days (IQR 1–5). Veno-venous extra corporal membrane oxygenation (VV-ECMO) was initiated in 7.4% of the mechanically ventilated patients, with a median duration of 10 days (IQR 6–21). Continuous renal replacement therapy (CRRT) because of acute kidney injury (AKI) was required in 10% of patients. The data on mechanical ventilation, proning, VV-ECMO and CRRT are shown in Table 4.

### 3.5. Antimicrobial Treatment

Oseltamivir was administered to 59.4% of the patients. Furthermore, antibiotic treatment was initiated in nearly all patients (94.8%), which can be attributed to the protocolized use of selective decontamination of the digestive tract (SDD). There was considerable variation in the antibiotics used, but the most commonly administered antibiotics were the cephalosporins ceftriaxone (51.1%), cefuroxime (27.1%), and cefotaxime (14.4%). Additionally, ciprofloxacin (38.3%) was frequently used, according to the national treatment guidelines for severe CAP. Although steroids are not routinely recommended in the treatment of severe viral CAP caused by influenza virus infections, patients were frequently treated with corticosteroids. Corticosteroids were administered to 60.4% of patients, primarily for exacerbation of COPD (44.5%), sepsis (25.6%), a corticosteroid stress protocol (7.6%), or severe community-acquired pneumonia (CAP) (6.0%). Mortality was higher in patients who received corticosteroids during ICU stay (79 (26.2%) vs. 30 (15.2%), *p* = 0.004); however, there were no differences in Aspergillus between these two groups (galactomannan positive 20 (6.6%) vs. 13 (6.6%) and culture 30 (10%) vs. 19 (9.6%)). Data on medication treatment are presented in Table 5.

### 3.6. Mortality and Length of Stay

The median ICU stay was 4 days (IQR 1–8), while total hospital stay was 10 days (IQR 6–21). Mortality was 21.9% in the ICU, and another 5.2% died after ICU discharge, totaling 27.1% hospital mortality. Factors independently associated with increased hospital mortality included age (OR = 1.039, *p* = 0.013), BMI (OR = 1.047, *p* = 0.044), having a neurodegenerative disorder (OR = 10.467, *p* = 0.006), having an autoimmune disease (OR = 5.555, *p* = 0.008), SAPS II score at admission (OR = 1.057, *p* < 0.0001), and escalation from non-invasive to invasive ventilation (OR = 2.224, *p* = 0.021).

## 4. Discussion

This study, examining the first post-COVID-19 pandemic influenza season in 34 ICUs in the Netherlands between November 2023 and March 2024, identified several important findings. First, mortality was substantial, with 21.9% of patients dying during ICU treatment. Second, bacterial superinfections were relatively common, with GAS identified in 20.3% of the patients with a bacterial co-infection. Third, this registry shows a relatively high percentage of patients with positive Aspergillus cultures, underscoring the well-established association between influenza and pulmonary aspergillosis [19]. Fourth, a higher BMI was independently associated with increased mortality, suggesting that obesity may influence disease severity in influenza, similar to findings in COVID-19 [19,20]. Lastly, a significant proportion of patients were young and without comorbidities, which warrants further comparison with the current 2024–2025 influenza season to assess any changes in disease patterns and trends post-COVID-10 pandemic.

Bacterial co-infections were common, with 37.6% of patients showing bacterial pathogens and thereby complicating treatment strategies. Specifically focusing on the high incidence of GAS. Knowledge of GAS prevalence facilitates early intervention considerations such as intravenous immunoglobulin (IVIG) and clindamycin to inhibit toxin production. The study also confirmed the significant detection of aspergillosis in critically ill patients with influenza, although the majority of mechanically ventilated patients was not tested, contrary to current guidelines. These findings underscore the necessity of prompt diagnosis and targeted therapy, including antifungal treatment, in managing critically ill influenza patients. It is important to note that there may be a risk of sampling bias in these results, as patients with more severe disease are more frequently sampled than those with less severe disease. Previous studies have reported co-infection rates ranging from 30% to 55% [11,21,22], this range underscores the challenge of diagnosing co-infection.

This study shows a substantial mortality rate among patients with influenza virus in the ICU. During ICU admission, 21.9% of patients died, and a total hospital mortality of 27.1% of patients died during their hospital stay. These mortality rates align with those reported in previous research on ICU patients with an influenza virus infection [21,23,24]. Certain factors may be associated with more severe disease and mortality, such as SAPS II scores and escalation from NIRS to IMV. Additionally, pre-existing comorbidities, including advanced age, neurodegenerative disorders, and autoimmune conditions, were also associated with higher mortality. Patients without comorbidities, often presented with more complicated disease in our cohort, evidenced by higher SAPS II scores, increased co-infections, and more frequent requiring interventions such as proning and mechanical ventilation. However, treatment restrictions may introduce bias that influences these results. Young, more healthy patients are less likely to face these treatment restrictions. In contrast, older patients or those with comorbidities may have limitations on their treatment options such as a do not intubate code. In our study, a do-not-resuscitate order was implemented in 38% of patients with comorbidities, compared to 13.3% of those without comorbidities. Similarly, a do-not-intubate order was implemented in 22.3% of patients with comorbidities, compared to 8.1% of patients without.

Steroid use was relatively high (60.4%), despite guidelines not recommending—or even contraindicating—the use of corticosteroids in patients with influenza infection. Corticosteroids may increase the risk of mortality and superinfections, particularly with *Aspergillus* species [25]. The high proportion of patients receiving corticosteroids in our cohort is likely attributable to other indications, such as exacerbation of COPD, sepsis, or severe CAP, rather than the influenza infection itself. There was no difference in the incidence of *Aspergillus* infection between patients who did and did not receive corticosteroids. However, mortality was higher in patients treated with corticosteroids compared to those who were not. This may, at least in part, be explained by the fact that patients with more severe illnesses were more likely to receive corticosteroids and already had an increased baseline risk of mortality prior to treatment because of comorbidities like COPD. These data from our retrospective cohort raise the question of whether corticosteroids are safe in patients with influenza; however, prospective studies are needed to confirm this.

Interestingly, this study identified an independent association between higher BMI and increased mortality. This finding contributes to the ongoing debate surrounding the obesity paradox, which suggests that (mild) obesity may be protective against severe outcomes in certain diseases [26]. For instance, previous studies have demonstrated a stronger association between obesity and increased mortality in patients with COVID-19 [19,20]. However, the role of obesity in patients with influenza remains controversial. While some evidence suggests it might contribute to more severe disease, clinical data on this relationship remain inconclusive [27,28,29]. Further research is needed to better understand the role of BMI and explore optimal treatment strategies, particularly in patients with obesity.

The percentage of patients requiring intubation and mechanical ventilation in this study is comparable to that reported previously [21,30,31] in which a median duration of IMV ranging from 5 to 10 days was reported [21,30,32,33], with more data supporting the latter duration. Thus, the median duration of 6 days observed in this study represents a relatively short period of ventilation for patients with an influenza virus. A possible explanation could be the prompt initiation of ventilation in prone position in a relatively large proportion of the patients. This could improve oxygenation and may facilitate a faster recovery. Compared to earlier studies, a relatively high proportion of patients were treated with prone positioning in this study (38%), whereas previous studies reported rates between 12% and 33% [30,33,34]. This approach may have been influenced by the recent COVID-19 pandemic, during which a substantial number of patients were ventilated in the prone position. As a result, clinicians and nurses have become more skilled in the procedure and recognizing the indication, potentially leading to a lower threshold for its implementation. However, as the comparability of the study population with previous research cannot be confirmed, this finding may reflect greater disease severity rather than changes in clinical practice.

This study has both strengths and limitations. One limitation is the challenge of determining whether a positive influenza RT-PCR test indicates the primary cause of pneumonia or an incidental finding. The study includes all ICU patients with a positive influenza RT-PCR test, meaning some patients may have been included whose primary illness is not influenza-related, introducing potential selection bias. Furthermore, influenza testing is not performed in every patient, leading to missed diagnosis and inclusions. Additionally, the retrospective design could introduce information bias, as data were extracted from clinical records by multiple researchers, which may have led to inconsistencies. Finally, only a few ICUs in the Netherlands are equipped for ECMO, a potential early intervention in severe respiratory failure due to influenza. Furthermore, some of the ECMO centers did not participate in this study, possibly underestimating the proportion of patients requiring ECMO. However, this study has several strengths;. It is one of the first studies to describe the post-COVID-19 seasonal influenza upsurge. The broad participation of ICUs nationwide enhances the generalizability of the findings and contributes to its validity by its large sample.

## 5. Conclusions

Between November 2023 and March 2024, patients with laboratory-confirmed influenza admitted to 34 participating Dutch ICUs were assessed for their disease characteristics and outcomes. This post-pandemic season was characterized by a high incidence of GAS infections complicating the treatment of influenza. A substantial group without comorbidities experienced more severe illness but had better survival rates. Further research is needed to explore additional therapeutic interventions, such as antiviral treatments, and to assess vaccination coverage and its effectiveness in ICU patients. Comparing the 2023–2024 data with future influenza seasons will be crucial for understanding trends and updating clinical practices.

## Figures and Tables

**Table 1:** Baseline characteristics and treatment limitations.

	*n* = 498
Age (years) [median (IQR)]	64 (55–72)
Sex [*n* (%) men]	293 (58.8)
BMI (kg/m^2^) [median (IQR)]	26.3 (22.1–30.9)
P/F ratio on admission ICU (mmHg) [*n* (%) yes]	
<80	43 (8.6)
80–100	52 (10.4)
100–200	204 (41.0)
200–300	116 (23.3)
>300	83 (16.6)
Comorbidities *n* (%)	
Cardiovascular	170 (34.1)
COPD	157 (31.5)
Diabetes	111 (22.3)
Respiratory other than COPD	89 (17.9)
Immunosuppressants	53 (10.6)
Chronic kidney failure	47 (9.4)
Solid malignancy	46 (9.2)
Autoimmune diseases	30 (6.0)
Hematologic malignancy	24 (4.8)
Neurodegenerative disorder	9 (1.8)
Smoking	178 (35.7)
SOFA-score [median (IQR)]	6 (3–9)
SAPS II-score [median (IQR)]	36 (27–48)
Leukocyte count, ×10^9^/L [median (IQR)]	10.5 (6.2–14.8)
C-reactive protein, mg/L [median (IQR)]	137 (58–276)
Duration of admission ICU (days) [median (IQR)]	4 (1–8)
Duration of admission hospital (days) [median (IQR)]	10 (6–21)
ICU mortality [*n* (%)]	109 (21.9)
Hospital mortality [*n* (%)]	135 (27.1)

Abbreviations: Interquartile range (IQR), Body mass index (BMI), partial pressure of arterial oxygen/fraction of inspired oxygen ratio (P/F-ratio), Chronic obstructive pulmonary disease (COPD), Sequential organ failure assessment score (SOFA-score), Simplified acute physiology II score (SAPS II-score), Intensive care unit (ICU).

**Table 2:** Bacterial co-infections and detection of *Aspergillus* species.

	*n* (%) Yes
Bacterial co-infection	187 (37.6)
Pathogen detected *n* = 187	*Streptococcus pneumoniae* 55 (31.1)
	*Group A Streptococcus, GAS* 36 (20.3)
	*Staphylococcus aureus* 15 (8.5)
	*Haemophilus influenzae* 13 (7.0)
	*Pseudomonas aeruginosa* 13 (7.0)
	*Klebsiella pneumoniae* 5 (2.7)
*Aspergillus fumigatus* grown in culture	49 (9.8)
*Aspergillus* galactomannan BAL fluid positive	Yes 9 (3.9), No 80 (34.9) Not tested 140 (61.1)
*Aspergillus* galactomannan sputum positive	Yes 30 (13.1) No 95 (41.5) Not tested 104 (45.4)

Only the mechanical ventilated patients were included in the *Aspergillus* culture and galactomannan analysis.

**Table 3:** Diagnostic imaging, laboratory results and severity of disease at admission to ICU.

	*n* = 498
Infiltrates on chest X-ray [*n* (%) yes]	304 (61.0)
Localisation [*n* (%) yes]	
Unilateral	122 (40.1)
Bilateral	174 (57.2)
Pleural effusion on chest X-ray [*n* (%) yes]	74 (14.9)
CT-scan within 72 h of admission [*n* (%) yes]	141 (28.4)
CT-scan findings [*n* (%)] *n* = 141	
Consolidation	117 (83.0)
Ground-glass opacity	49 (34.7)
Pleural effusion	35 (24.8)
Tree-in-bud	13 (9.2)
Fibrosis	4 (2.8)
Traction bronchiectasis	4 (2.8)
Pneumothorax	2 (1.4)

Abbreviations: Intensive care unit (ICU), Computer tomography (CT), Sequential organ failure assessment score (SOFA-score), Interquartile range (IQR), Simplified acute physiology II score (SAPS II-score), partial pressure of arterial oxygen/fraction of inspired oxygen ratio (P/F-ratio).

**Table 4:** Non-invasive and invasive respiratory support, ECMO and renal replacement therapy.

NIRS at admission ICU [*n* (%) yes]	295 (59.2)
Type NIRS [*n* (%)]	HFOT 171 (58.6) CPAP 36 (12.3) BiPAP 79 (27.1)
Duration of NIRS days [median (IQR)]	1 (1–2)
Escalation to IMV [*n* (%) yes]	108 (36.7)
Timing of escalation days [median (IQR)]	1 (0–2)
P/F-ratio in case of escalation to IMV (mmHg)	
<80	27 (22.7)
80–100	19 (16.0)
100–200	46 (38.7)
200–300	16 (13.4)
IMV at admission [*n* (%) yes]	121 (32.7)
Patients on IMV during ICU course [*n* (%) yes]	229 (46.0)
Duration of IMV days [median (IQR)]	6 (3–17)
P/F ratio at start of IMV, mmHg [*n* (%)]	
<80	35 (20.0)
80–100	21 (12.0)
100–200	84 (48.0)
200–300	22 (12.6)
Lowest P/F-ratio during ICU stay [median (IQR)]	119 (83–179)
Highest PaCO_2_, mmHg [median (IQR)]	55.1 (44.1–70.0)
Highest PEEP, cmH_2_O [median (IQR)]	12 (9–14)
Highest driving pressure, cm H20 [median (IQR)]	20 (14.0–28.0)
Proning [*n* (%) yes]	87 (38.0)
P/F-ratio at start proning [*n* (%)]	
<80	32 (34.4)
80–100	20 (21.5)
100–200	29 (31.2)
200–300	0
Days between intubation and start proning [median (IQR)]	0 (0–1)
Duration of proning (days) [median (IQR)]	2 (1–5)
VV-ECMO [*n* (%) yes]	17 (7.4)
P/F ratio at start VV-ECMO	
<80	8 (47.1%)
80–100	4 (23.5)
100–200	2 (11.8)
200–300	1 (5.9)
Days between intubation and start VV-ECMO [median (IQR)]	1 (0–3)
Duration of ECMO (days) [median (IQR)]	10 (6–21)
CRRT [*n* (%) yes]	50 (10.0)

Table 4 presents the number of patients who underwent non-invasive respiratory support (NIRS) and invasive mechanical ventilation (IMV) along with the duration of respiratory support. Additionally, it provides the measured parameters of ventilation. Proning and VV-ECMO are reported exclusively for mechanically ventilated patients. Abbreviations: Noninvasive respiratory support (NIRS), High flow oxygen therapy (HFOT), Continuous positive airway pressure (CPAP), Bilevel positive airway pressure (BiPAP), Invasive mechanical ventilation (IMV), Interquartile range (IQR), partial pressure of arterial oxygen/fraction of inspired oxygen ratio (P/F ratio), positive end-expiratory pressure (PEEP), veno-venous extracorporeal membrane oxygenation (VV-ECMO), Continuous renal replacement therapy (CRRT).

**Table 5:** Treatment with corticosteroids, antibacterial and antiviral drugs.

	*n*(%) yes
Corticosteroids	201 (60.4)
Indication for corticosteroids	
Exacerbation COPD	134 (44.5%)
Sepsis	77 (25.6%)
Corticosteroid stress protocol	23 (7.6)
Severe CAP	18 (6.0)
ARDS	16 (5.3)
Exacerbation asthma	7 (3.5)
Other	20 (10.0)
Days start after ICU admission [median (IQR)]	0 (0–1)
Antibiotic treatment	472 (94.8)
Type antibiotic	
Cephalosporins	437 (92.6)
Fluoroquinolones	194 (41.1)
Aminoglycosides	29 (6.1)
Penicillins	25 (5.3)
Tetracyclines	16 (3.4)
Macrolides	10 (2.1)
Carbapenems	9 (1.9)
Other	26 (5.5)
Oseltamivir	296 (59.4)

Table 5 presents the frequency and type of used medications, including corticosteroids, antibiotics and antiviral medications. Other for corticosteroids included: thyrotoxic crisis, myasthenic crisis, lung fibrosis and cryptogenic organizing pneumonia. Other for antibiotics included co-trimoxazole and metronidazole. Abbreviations: Chronic Obstructive Pulmonary Disease (COPD), Community Acquired Pneumonia (CAP), Acute Respiratory Distress Syndrome (ARDS), Intensive Care Unit (ICU).

## Data Availability

Data will be available to the contact author upon reasonable request.

## Appendix A

**Table A1:** List of Participating Intensive Care Units.

Adriaan de Ruijter Ziekenhuis, Goes
Albert Schweitzer Ziekenhuis, Dordrecht
Alrijne Ziekenhuis, Leiderdorp
Amphia, Breda
Bravis Ziekenhuis, Bergen op Zoom
Catharina Ziekenhuis, Eindhoven
Diakonessenhuis, Utrecht
Dijklander Ziekenhuis, Hoorn/Purmerend
Elisabeth-TweeSteden Ziekenhuis, Tilburg
Erasmus Medisch Centrum, Rotterdam
Flevoziekenhuis, Almere
Franciscus Gasthuis & Vlietland, Rotterdam
Fries Medisch Centrum (Frisius MC), Heerenveen
Gelre Ziekenhuizen, Apeldoorn
Groene Hart Ziekenhuis, Gouda
HagaZiekenhuis, Den Haag
Ikazia Ziekenhuis, Rotterdam
IJsselland Ziekenhuis, Capelle aan den IJssel
Isala, Zwolle
Jeroen Bosch Ziekenhuis, ’s-Hertogenbosch
Martini Ziekenhuis, Groningen
Maasstad Ziekenhuis, Rotterdam
Máxima Medisch Centrum, Veldhoven
Meander Medisch Centrum, Amersfoort
Medisch Centrum Leeuwarden, Leeuwarden
Noordwest Ziekenhuisgroep
Onze Lieve Vrouwe Gasthuis (OLVG), Amsterdam
Reinier de Graaf Groep, Delft
Rijnstate, Arnhem
Spaarne Gasthuis, Haarlem/Hoofddorp
St. Antonius Ziekenhuis, Nieuwegein
Tergooi Medisch Centrum, Hilversum/Blaricum
UMC Groningen (UMCG), Groningen
Zaans Medisch Centrum, Zaandam
